# Physiological traits and related gene expression of salt tolerance in hybrid rice *Jingliangyou 3261* at the seedling stage

**DOI:** 10.1186/s12870-026-08891-2

**Published:** 2026-05-06

**Authors:** Xiabing Sheng, Donger Zhang, Jie Tang, Yuanyi Hu, Zhigang Zhang, Dan Zhang, Xiayu Guo, Huimin Liu, Aibin He, Yishan Yang, Zhiyong Ai, Yingjiang Li, Xiaolin Liu

**Affiliations:** 1 National Center of Technology Innovation for Saline-Alkali Tolerant Rice in Sanya, Sanya, Hainan China; 2 Hunan Hybrid Rice Research Center, Key Laboratory of Saline-Alkali Tolerant Rice Biology and Genetic Breeding, Ministry of Agriculture and Rural Affairs, Changsha, Hunan China

**Keywords:** Hybrid Rice, *Jingliangyou3261*, Salt tolerance, Physiological traits

## Abstract

**Background:**

Soil salinity severely restricts rice growth and productivity, particularly at the seedling stage when plants are highly sensitive to ionic and osmotic stress.

**Results:**

In this study, we systematically evaluated the phenotypic, physiological, and molecular responses of the hybrid rice cultivar *Jingliangyou 3261* under salt stress. Compared with the control cultivars (*Yuxiangyouzhan*,* Huazhan*,* and Jingliangyouhuazhan*), *Jingliangyou 3261* seedlings exhibited significantly higher survival rates, less leaf chlorosis, and stronger recovery after stress relief. Physiological analyses indicated smaller declines in chlorophyll content, reduced malondialdehyde (MDA) accumulation, and enhanced synthesis of soluble proteins. Moreover, *Jingliangyou 3261* maintained superior ion homeostasis by limiting Na⁺ accumulation and sustaining higher K⁺ levels, resulting in a relatively stable Na⁺/K⁺ ratio. Antioxidant capacity was enhanced, as reflected by elevated superoxide dismutase (SOD) and peroxidase (POD) activities together with reduced reactive oxygen species (ROS) accumulation. Molecular analysis showed that under salt stress, osmotic adjustment genes (*OsP5CS1/2*, *OsLEA3*), ion transport genes (*OsHKT*, *OsNHX*, *OsHAK*, *OsKAT*, *OsSOS1*), and antioxidant-related genes (*OsCSD*, *OsCAT*, *OsAPX*) were significantly up-regulated in *Jingliangyou 3261*.

**Conclusions:**

These coordinated physiological and molecular responses provide a comprehensive mechanistic basis for the superior salt tolerance of *Jingliangyou 3261.* Our findings not only deepen the understanding of salt tolerance mechanisms in hybrid rice but also provide valuable genetic resources for salt-tolerant breeding and the utilization of saline–alkali soils.

## Instruction

Rice (*Oryza sativa* L.) is a staple crop for over half of the world’s population, playing a crucial role in global food security [1, 2]. With continuous population growth and shrinking arable land, utilizing saline-alkali lands and improving rice yield under adverse conditions have become critical challenges [3]. The seedling stage is particularly sensitive to salt stress, which inhibits growth through ion toxicity, osmotic stress, and oxidative damage, thereby threatening seedling establishment and final yield [4]. Therefore, understanding the physiological and molecular mechanisms of salt tolerance at the seedling stage is essential for breeding salt-tolerant varieties [5].

Salt stress reduces plant height, root length, and leaf area, and accelerates senescence [6]. It decreases chlorophyll content and photosynthetic rate, impairing energy supply and material synthesis [7]. To cope, plants accumulate compatible solutes such as proline, trehalose, glycine betaine to lower cellular osmotic potential and restore water uptake [8, 9]. A key indicator of salt tolerance is a low Na⁺/K⁺ ratio, achieved by selective K⁺ uptake and Na⁺ compartmentalization [10].

Excessive reactive oxygen species (ROS) cause oxidative damage to lipids, proteins, and DNA. Plants deploy enzymatic and non-enzymatic antioxidant systems to scavenge ROS and maintain redox homeostasis [11]. The enzymatic system includes superoxide dismutase (SOD), peroxidase (POD), catalase (CAT), and ascorbate peroxidase (APX); the non-enzymatic system includes ascorbic acid (AsA) and glutathione (GSH) [12]. Under salt stress, proline biosynthesis genes (*OsP5CS1*,* OsP5CR*) are upregulated, enhancing osmotic regulation [13, 14]. Late embryogenesis abundant (LEA) proteins, such as *OsLEA3-2* and *OsLEA4*, stabilize cell membranes and regulate osmotic balance, directly contributing to salt tolerance [15, 16].

Ion homeostasis is maintained by Na⁺ transporters (high-affinity K⁺ transporter, HKT family) and non-selective cation channels (NSCC). *OsHKT1;1* restricts long-distance Na⁺ transport from roots to shoots by mediating Na⁺ transfer from xylem to parenchyma cells and promoting Na⁺ recirculation from shoots to roots, thereby enhancing shoot salt tolerance [17, 18]. The plasma membrane Na⁺/H⁺ antiporter Salt-Overly Sensitive 1 (SOS1), activated via the *OsCBL4*-*OsCIPK24* pathway, mediates root Na⁺ efflux, while vacuolar Na⁺/H⁺ exchangers (NHX) sequester Na⁺ into vacuoles [19, 20]. K⁺ uptake and homeostasis are maintained by *OsHAK*, *OsAKT1*, and *OsKCO1* transporters; overexpression of *OsNHX1* reduces cytosolic Na⁺ and improves salt tolerance [21, 22].

Salt stress activates ROS as signaling molecules, requiring a balance between signaling and toxicity [23, 24]. In rice, a hierarchical antioxidant system operates: SOD converts O₂⁻ to H₂O₂ [25, 26]; APX scavenges H₂O₂ [27, 28]; and the GR/GRX pathway regenerates GSH [29, 30]. This coordinated defense maintains redox homeostasis and protects membrane integrity under salt stress.

Despite the development of several salt-tolerant rice varieties, their performance in saline-alkali lands remains insufficient for large-scale promotion. *Jingliangyou 3261* is a new salt-tolerant indica two-line hybrid rice jointly developed by the National Center of Technology Innovation for Saline-Alkali Tolerant Rice and the Hunan Hybrid Rice Research Center, exhibiting strong salt tolerance, good grain quality, and robust resistance. However, its salt-tolerance mechanism at the seedling stage is unclear. In this study, four rice varieties—*Jingliangyou 3261*, *Yuxiangyouzhan*, *Huazhan*, and *Jingliangyouhuazhan*—were treated with 0.8% salt stress to analyze morphological, physiological, and gene expression changes. The aim is to elucidate the seedling-stage salt tolerance mechanism of *Jingliangyou 3261*, providing theoretical references for salt-tolerant rice breeding.

## Plant materials and methods

### Plant material and growth conditions

This study employed the salt-tolerant hybrid rice cultivar *Jingliangyou 3261* (National Approval No. Guoshen Rice 20244002, Xiangshendao 20241003) as the primary experimental material. Three additional cultivars were selected as multidimensional controls with distinct rationales: (1) *Yuxiangyouzhan* (Qiongshendao 2007015), a widely planted conventional aromatic indica rice with excellent grain quality and broad adaptability in South China coastal regions, serving as a representative moderately salt-sensitive conventional control; (2) *Huazhan*, a classic elite backbone restorer line with strong wide adaptability, which is a core male parent germplasm widely used in commercial hybrid rice breeding in China; and (3) *Jingliangyouhuazhan* (Guishendao 2019192), a sibling hybrid combination that shares the identical male parent (Huazhan) and a closely related female pedigree with Jingliangyou 3261, used to exclude genetic background interference and specifically evaluate the unique salt-tolerance superiority of the tested variety.

Seedling‑stage salt stress experiments were conducted using hydroponic culture. A 0.8% NaCl solution (approximately 137 mM) was selected for salt stress treatment. This concentration effectively induces physiological salt tolerance responses, clearly distinguishes varietal differences in salt tolerance, and represents a common standard concentration in rice salt tolerance research, ensuring the reproducibility and comparability of experimental data.Seeds were surface‑sterilized, soaked, and germinated. At the one‑leaf‑one‑heart stage, seedlings were transferred to Yoshida nutrient solution (pH 5.7). At the three‑leaf stage, the experimental group was subjected to salt stress by adding 0.8% NaCl to the Yoshida nutrient solution for 12 days, while the control group was maintained in salt‑free nutrient solution throughout. After the salt treatment, the experimental group was returned to salt‑free nutrient solution for a 7‑day recovery period. Nutrient solutions were renewed every 3 days throughout the cultivation period to maintain nutrient stability.Samples were collected at 0, 1, 3, 6, 9, and 12 days after salt treatment, as well as on the 7th day of recovery, for phenotypic observations. All samples were stored at ‑80 °C for subsequent physiological and biochemical analyses.

### Phenotype analysis and evaluation of salinity tolerance

Measure seedling height and primary root length using a ruler. Acquire root images using a root scanner (ScanMakeri800plus, Zhejiang, China) and quantify topological parameters such as root volume, surface area, mean diameter, projected area, and root tip count via a root analysis system (GXY-A, Zhejiang, China). Fresh weights of stems, leaves, and roots were weighed using an electronic balance. After drying to constant weight, dry weights of each part were determined. Survival rate was defined as the presence of newly emerged green leaves during recovery cultivation [31]. Modified from previous formulas, relative loss rate (RLR) and relative growth rate (RGR) were introduced to quantify salt stress effects [32].

Total Fresh Weight (g) = Aboveground Fresh Weight + Root Fresh Weight.

Total Dry Weight (g) = Aboveground Dry Weight + Root Dry Weight.

Survival Rate (%) = (Number of Surviving Plants / Total Number of Observed Plants) × 100%.

Relative Loss Rate/Growth Rate (%)

$$=\:\left\{\begin{array}{c}\frac{\text{Control Group}\:-\:\text{Treated Group}}{\text{Control Group}}\:\times\:100\%,\:\text{if Control}\:\ge\:\mathrm{Treated}\\ \frac{\text{Treated Group}\:-\:\text{Control Group}}{\text{Control Group}}\:\times\:100\%,\:\text{if Control}\: < \:\mathrm{Treated}\end{array}\right.$$

### Measurement of physiological indicators

The chlorophyll content in rice leaves was determined using a plant chlorophyll content assay kit (ZCIBIO, Shanghai, China) to measure Chlorophyll (Chl), Chlorophyll a (ChlA), and Chlorophyll b (ChlB) levels. Using scissors, finely chop 0.1 g of rice leaves and place them in a 15 mL centrifuge tube. Add 1 mL of distilled water and 0.05 g of a calcium carbonate and quartz sand mixture, then grind thoroughly. Extract the mixture using a solvent mixture (anhydrous ethanol: acetone = 1:2). Centrifuge tubes were incubated in darkness for 3 h until plant tissue residues turned completely white. The supernatant was collected and measured for absorbance at 663 nm, 645 nm, and 470 nm using a microplate reader to calculate chlorophyll content, Each sample was analyzed in triplicate.

### Determination of MDA content

The determination of malondialdehyde (MDA) was performed using the Thiobarbituric acid method (TBA) [33]. Take 0.1 g of plant sample ground in a mill, add 1 mL of extraction solution, mix thoroughly, and centrifuge. Take 100 µL of the supernatant and mix with 100 µL of distilled water and 300 µL of TBA working solution (0.5% TBA + 10% Trichloroacetic acid (TCA). React the mixture in a boiling water bath for 60 min. After centrifugation, the supernatant was collected and measured for absorbance at 532 nm, 600 nm, and 450 nm using a microplate reader, Each sample was analyzed in triplicate.

### Determination of soluble protein content

Soluble protein content was determined using the Coomassie Brilliant Blue G-250 method [34]. Weigh 0.1 g of plant sample, grind it into powder, add 1 mL of extraction solution, mix thoroughly, centrifuge, and collect the supernatant. Take 100 µL of the supernatant, mix with an appropriate amount of Bradford working solution, react at room temperature for 5 min, and measure the absorbance at 595 nm. Plot a standard curve using bovine serum albumin (BSA) standard solutions. Calculate the soluble protein content in the sample based on the absorbance values, Each sample was analyzed in triplicate.

### Assay of SOD and POD activity

Weigh 0.1 g of sample, add 1 mL of pre-chilled extraction solution, grind in an ice bath, then centrifuge at 4 °C and 12,000 g for 20 min. Remove the supernatant and measure SOD and POD activities according to the protocols established by Kim et al. [35]. Measurements of absorbance at 560 nm and 470 nm were performed using a microplate reader (BioTec Synergy LX, Synergy Corporation, Houston, TX, USA) to analyze SOD and POD activity in the samples, Each sample was analyzed in triplicate.

### Measurement of Na^+^ and K^+^ contents

Separate the aboveground parts (stems and leaves) from the underground parts (root system) of rice seedlings. Wash three times with ultra-pure water using a vortex to remove surface contaminants. Subsequently, subject the samples to heat inactivation at 105 °C for 30 min to completely deactivate enzyme activity, then transfer them to an 80 °C oven and dry to constant weight. Additionally, 0.2 g of the dried sample was digested with 6 mL of HNO₃ for 2 h, further diluted with 2 mL of deionized water, and then subjected to microwave digestion at 180 °C for 15 min (MARS XPRESS, MBE Corporation, Matthews, NC, USA). Na^+^ and K^+^ content were measured using inductively coupled plasma-MS (Thermo Fisher Scientific iCAP PQ, Waltham, MA, USA) [36], Each sample was analyzed in triplicate.

$$\triangle\;\mathrm{Na}^+/\;\mathrm{K}^+=(\mathrm{Na}^+/\;\mathrm{K}^+)\;0.8\%\;\mathrm{NaCl}-(\mathrm{Na}^+/\;\mathrm{K}^+)0\%\mathrm{NaCl}$$

### Histochemical staining

Detect cellular accumulation of O₂⁻ and H₂O₂ using nitroblue tetrazolium (NBT) and 3,3′-diaminobenzidine (DAB) histochemical staining [37, 38]. Isolated leaves were immersed in NBT and DAB staining solutions (Coolaber Technology, Beijing, China), evacuated at -0.1 MPa for 30 min, and incubated in the dark at 25 °C for 8 h. After staining, samples were decolorized with 95% ethanol in an 80 °C water bath until chlorophyll was completely removed and the background became transparent. Decolorized samples were examined and photographed under an optical microscope. Positive NBT-stained regions appear as blue spots, while positive DAB-stained regions appear as brown spots. Non-stress control groups were established to exclude non-specific staining, Each sample was analyzed in triplicate.

### Determination of salt tolerance gene expression levels

Total RNA was extracted from various rice tissues using a total RNA extraction kit (Vazyme, Jiangsu, China). cDNA was synthesized using a kit (Vazyme, Jiangsu, China) with 1 µg of total RNA as template. Quantitative real-time PCR (qRT-PCR) was performed using the LightCycler 480 system (Roche Diagnostics, Rotkreuz, Switzerland). Relative expression levels of selected genes were calculated using the 2^−ΔΔCT^ method [39], with *OsActin* as the internal reference gene. Genes and primers used for qRT-PCR are detailed in Table 1. Each sample was analyzed in triplicate.

**Table 1:** Primer sequences used in qRT-PCR

Gene name	LOC number	primersequence
*OsActin*	LOC_Os03g50885	F: CCCAGATCATGTTTGAGACC R: TGAATGAGTAACCACGCTCC
*OsP5CS1*	LOC_Os05g38150	F: GGTCAGAGTGGACTGATGGC R: CCCGGAACTTTGGGTTCTCA
*OsP5CS2*	LOC_Os01g62900	F: GCAGAACCCACAGATGGACA R: CTCCTGGTACTGATGGCGTC
*OsLEA3*	LOC_Os05g46480	F: AATGATTTCCCTTTGGGTC R: CATCAGTACACATCACCCA
*OsHKT1;1*	LOC_Os04g51820	F: ATTAGCAGAGCACTGTGGAGGAA R: CCGACGAACCCGTAGGAAG
*OsHKT1;4*	LOC_Os04g51830	F: CCTACCTGACCATCTTTGTCAT R: TCTCCCTACGAAACCAGTCCA
*OsHKT1;5*	LOC_Os01g20160	F: TGCCACCTTACACCACTTTCG R: TGCCATACGCACTGATAACCTC
*OsNHX1*	LOC_Os07g47100	F: GGTGGCTGAGTTGCTAGATTTGA R: GGATTTGCCAGGTCTGTCACT
*OsNHX2*	LOC_Os11g42790	F: GCAAGTAATCATATGGTGGGCAG R: GTGGGTCTATGATTGACTGGCTT
*OsNHX4*	LOC_Os06g21360	F: CTTTGGAATCATCATATCGCTCG R: ATTGTACTGGTGATGATGGTTGC
*OsNHX5*	LOC_Os09g11450	F: TTATCGGAGGATCGACGGGCA R: GTCAGATAGTTCCTGTCAAGGGC
*OsHAK1*	LOC_Os04g32920	F: GTTGATGATGCTGATGTTGGAAG R: CCAACACTTTCAGCTGAAAC
*OsHAK7*	LOC_Os07g47350	F: TGCTGTGACACTTGGTTTCC R: AAATAACAAGGCGAGCAGGA
*OsKAT3*	LOC_Os02g14840	F: ATCTTGCTTCCACCAGTTCTGG R: ACTAGGACTATAAGGAACAGCT
*OsSOS1*	LOC_Os12g44360	F: GACACTGGGAATGTTCTATGC R: CCATGAAGCACCGTGCCTCTCG
*OsCSD1*	LOC_Os03g22810	F: TTGGAAATGTCACCGCTGGA R: TTTCCGGTGGTCTTGCTCAG
*OsCSD2*	LOC_Os07g46990	F: GACCTCTGTGACGGGAAGTG R: AGTAAGGGGGATCTGGCTGT
*OsCATA*	LOC_Os02g02400	F: CCCCGAGTGGAAGCTGTT R: CGACGTTGCGGTTGAGAA
*OsCATB*	LOC_Os06g51150	R: AGTTTGACAGGGAGCGTAT R: GGTCTGAACACCAGGAGC
*OsAPX1*	LOC_Os03g17690	F: ACCCACCATCTCCTACGC R: TGCAGGTITGTCCTCCCT
*OsAPX2*	LOC_Os07g49400	F: TTGTGAGTGGCGAGAAGGA R: GGCGTAATCCGCAAAGAA

### Statistical analysis

Data organization and basic statistical analysis were performed using Excel 2019. One-way analysis of variance (ANOVA) was conducted using SPSS 26.0. When results were significant, multiple comparisons and tests for significant differences were further performed using the LSD method. graphs were generated using GraphPad Prism 8.

## Results

### Morphological and phenotypic responses of rice seedlings to salt stress

**Fig. 1:**
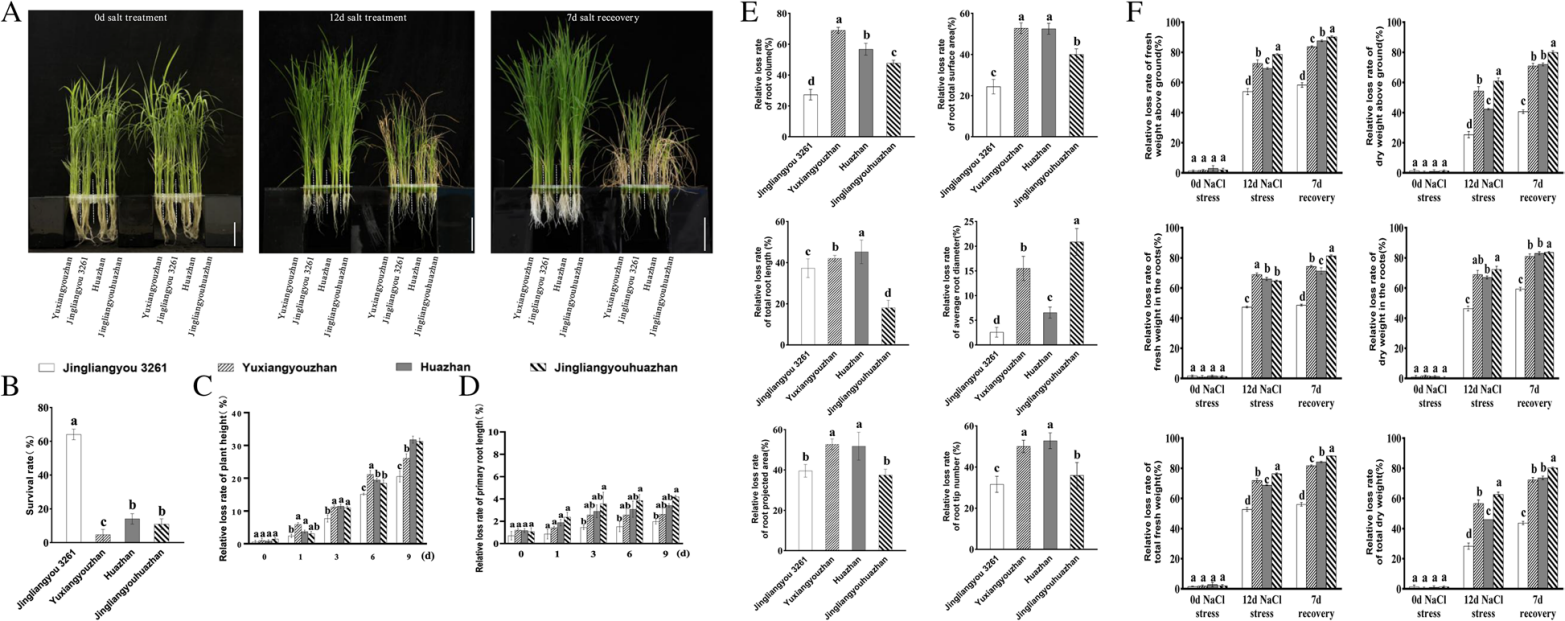
Variations in seedling salt tolerance and growth-related traits among different rice cultivars under salt stress and recovery conditions. **A** Phenotypic performance of rice cultivars *Jingliangyou 3261*, *Yuxiangyouzhan*, *Huazhan* and *Jingliangyouhuazhan* following 12-day salt treatment and subsequent 7-day recovery. **B** Survival rate of materials different materials after salt stress. **C** Relative plant height of different materials under 0, 1, 3, 6, and 9 days of salt treatment. **D** relative root length of different materials under 0, 1, 3, 6, and 9 days of salt treatment. **E** Relative loss rates of root morphological traits in different rice cultivars under salt stress. **F** Relative loss rates of biomass and organ weights in different rice cultivars under salt stress and recovery conditions. Data are presented as mean ± SE of three independent biological replicates (*n* = 3). Different lowercase letters denote significant differences among treatments at each time point (Duncan’s multiple range test, *P* < 0.05). Scale bars are 5 cm

Salt stress markedly inhibited seedling growth, and the inhibitory effects intensified with the extension of salt treatment duration. After 12 days of salt exposure, root growth was severely restricted and leaf chlorosis and wilting were prominent in *Yuxiangyouzhan*, *Huazhan*, and *Jingliangyouhuazhan*. In contrast, *Jingliangyou 3261* showed much stronger tolerance, with relatively mild chlorosis and a more developed root system. After 7 days of recovery, the leaves of the control varieties further withered, whereas *Jingliangyou 3261* regained leaf greenness and exhibited better root growth (Fig. 1A). The survival rate of *Jingliangyou 3261* was 64.06%, which was significantly higher than those of *Yuxiangyouzhan* (4.69%, *P* < 0.01), *Huazhan* (14.06%, *P* < 0.01), and *Jingliangyouhuazhan* (10.94%, *P* < 0.01) (Fig. 1B).

Plant height (Fig. 1C) and primary root length (Fig. 1D) decreased significantly in all cultivars under 0.8% NaCl treatment (*P* < 0.05), but the reductions of reduction was consistently milder in *Jingliangyou 3261*. Root system traits such as root volume, total surface area, and tip number also declined under salt stress (Fig. 1E), yet the degree of inhibition was relatively lower in *Jingliangyou 3261* compared with the three control cultivars. Although biomass accumulation was reduced in both shoots and roots in all cultivars (Fig. 1F), *Jingliangyou 3261* maintained higher relative values, suggesting enhanced physiological adaptability.

### Photosynthetic pigments and oxidative stress responses under salt stress

**Fig. 2:**
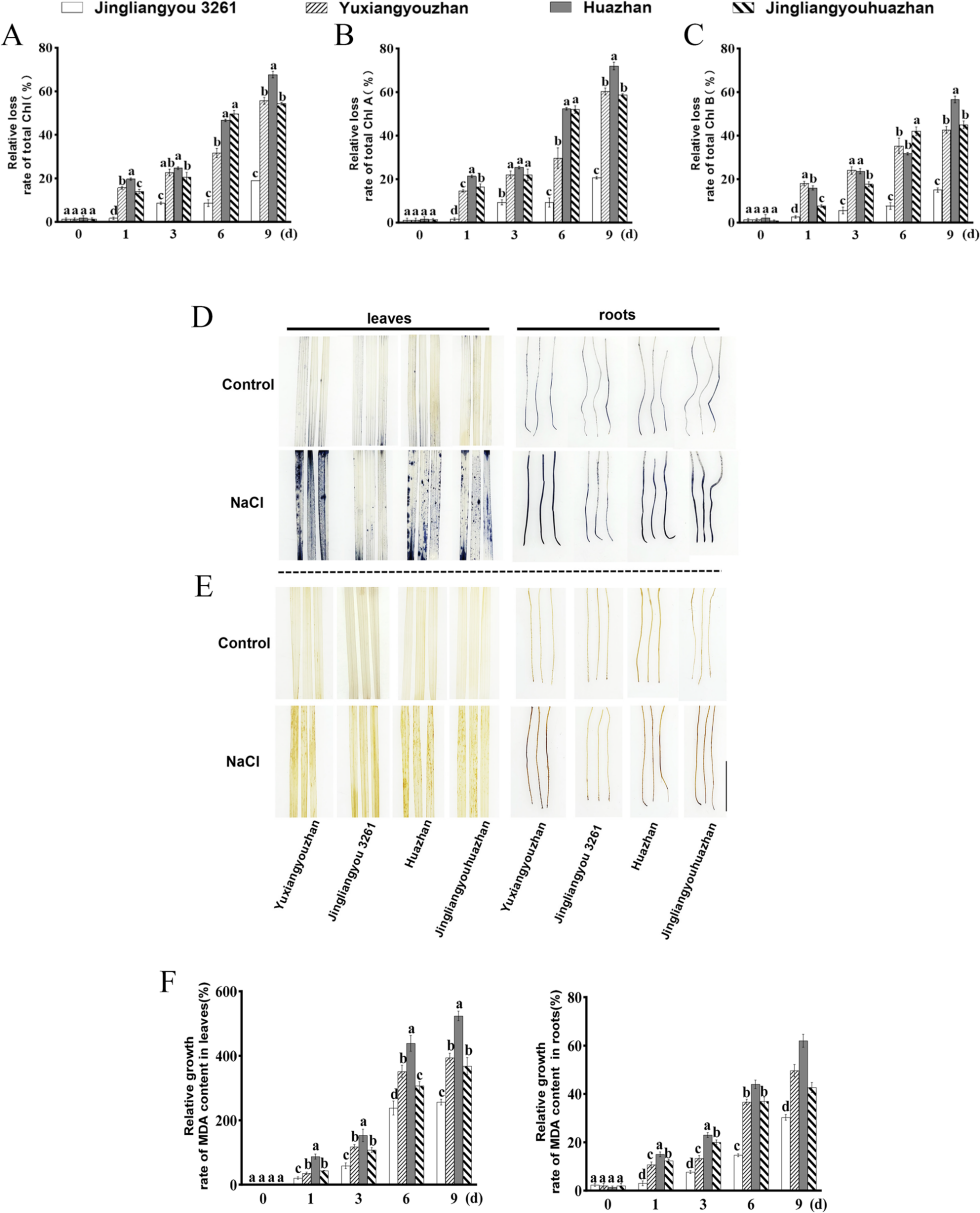
Photosynthetic pigment content and oxidative stress responses of contrasting rice cultivars under prolonged salt stress. **A** Total chlorophyll content in different rice cultivars at 0, 1, 3, 6, and 9 days under salt stress. **B** Chlorophyll a content in different rice cultivars at 0, 1, 3, 6, and 9 days under salt stress. **C** Chlorophyll b content in different rice cultivars at 0, 1, 3, 6, and 9 days under salt stress. **D** NBT staining showing superoxide anion (O₂⁻) accumulation in rice leaves and roots under salt stress. **E** DAB staining showing hydrogen peroxide (H₂O₂) accumulation in rice leaves and roots under salt stress. **F** Malondialdehyde (MDA) content in leaves and roots of rice at 0, 1, 3, 6, and 9 days under salt stress. Data are presented as mean ± SE of three independent biological replicates (*n* = 3). Different lowercase letters denote significant differences among treatments at each time point (Duncan’s multiple range test, *P* < 0.05). Scale bars are 1 cm

Chlorophyll content progressively decreased in all cultivars under prolonged salt stress. However, the decreases in total chlorophyll (*P* < 0.01) (Fig. 2A), chlorophyll a (*P* < 0.01) (Fig. 2B), and chlorophyll b (*P* < 0.01) (Fig. 2C) were significantly lower in *Jingliangyou 3261*, suggesting superior maintenance of photosynthetic capacity.

NBT (Fig. 2D) and DAB (Fig. 2E) staining showed that *Jingliangyou 3261* accumulated less O₂⁻ and H₂O₂ in both leaves and roots compared with the controls. Consistently, although MDA content increased progressively in all cultivars under salt stress, the levels in *Jingliangyou 3261* remained significantly lower throughout the treatment (*P* < 0.01). After 12 days of salt stress, leaf and root levels increasing by 256.35% and 30.16%, markedly lower than in the other varieties (*P* < 0.01) (Fig. 2F).These results indicate that *Jingliangyou. 3261* suffers less lipid peroxidation and oxidative damage under salinity.

### Soluble protein accumulation and ion homeostasis under salt stress

**Fig. 3:**
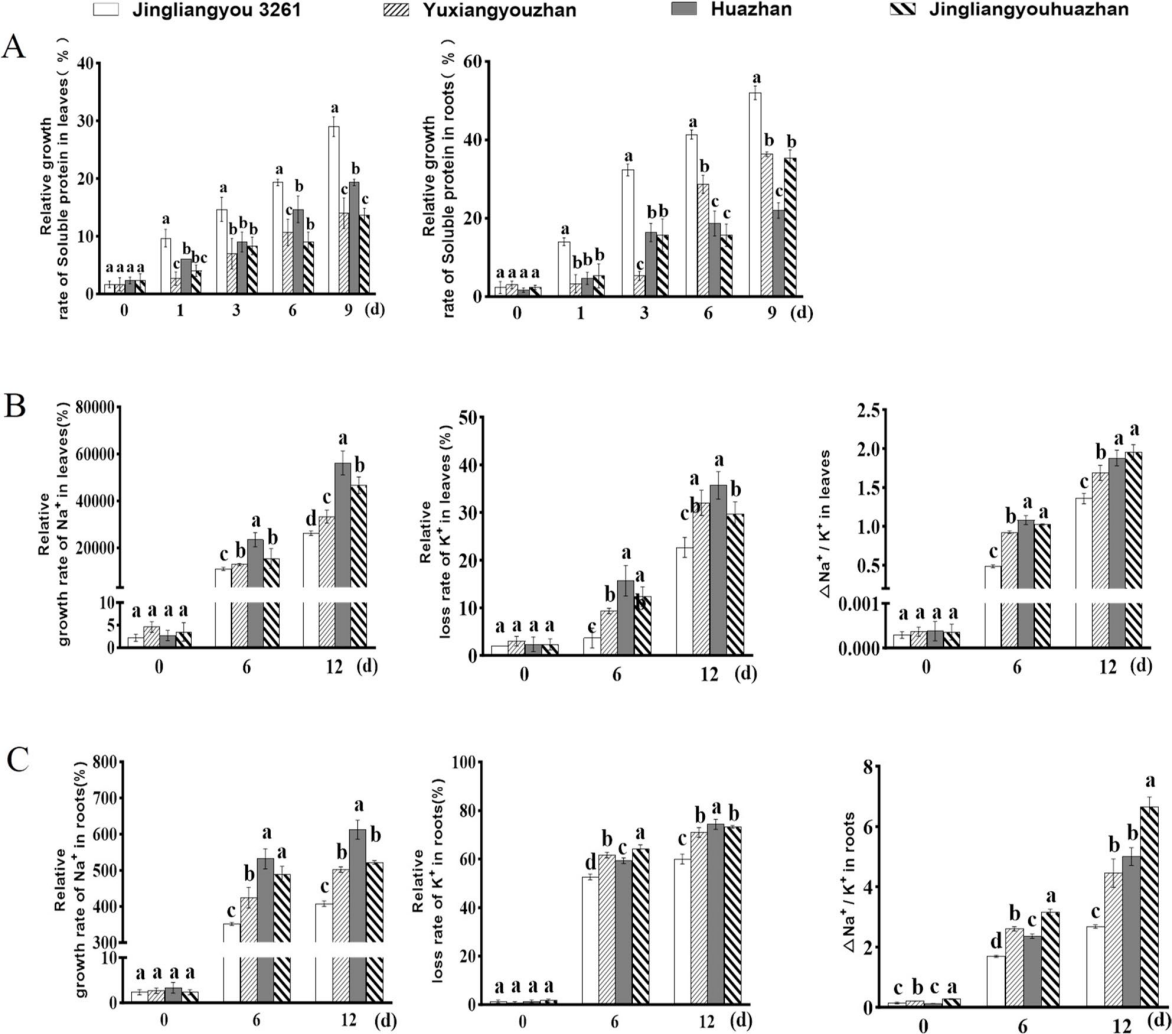
Soluble Protein Accumulation and Ion Homeostasis Responses of Contrasting Rice Cultivars Under 0.8% Salt Stress. **A** Soluble protein content in leaves and roots of different rice cultivars at 0, 1, 3, 6, and 9 days under 0.8% salt stress. **B** Na⁺ accumulation in leaves and roots of different rice cultivars after 0, 6, and 12 days of 0.8% salt stress. **C** Na⁺/K⁺ ratio in leaves and roots of different rice cultivars after 0, 6, and 12 days of 0.8% salt stress.Data are presented as mean ± SE of three independent biological replicates (*n* = 3). Different lowercase letters denote significant differences among treatments at each time point (Duncan’s multiple range test, *P* < 0.05)

Under 0.8% salt stress, soluble protein levels increased in both leaves and roots of all cultivars, with *Jingliangyou 3261* showing the largest increments. By day 9, leaf soluble protein increased by 29.21% in *Jingliangyou 3261*, significantly higher than in the three control cultivars (13.85%–19.37%,*P* < 0.01). Root soluble protein content also increased more strongly in *Jingliangyou 3261* than in the other varieties, suggesting enhanced stress-related protein synthesis (Fig. 3A).

Salt stress induced pronounced Na⁺ accumulation and K⁺ depletion in all cultivars, but these responses were attenuated in *Jingliangyou 3261*. By day 12, Na⁺ content in leaves and roots of *Jingliangyou 3261* increased by 26,195.34% and 407.46%, respectively, values significantly lower than those of the controls (*P* < 0.01). Meanwhile, K⁺ loss was mitigated, with reductions of 22.52% in leaves and 59.76% in roots, compared with 30.00–74.00% in the other varieties (*P* < 0.01) (Fig. 3B, C). As a result, the increase in the Na⁺/K⁺ ratio was markedly alleviated in *Jingliangyou 3261*, confirming its superior ability to maintain ionic homeostasis under salt stress.

### Alterations in antioxidant enzyme activities under salt stress

**Fig. 4:**
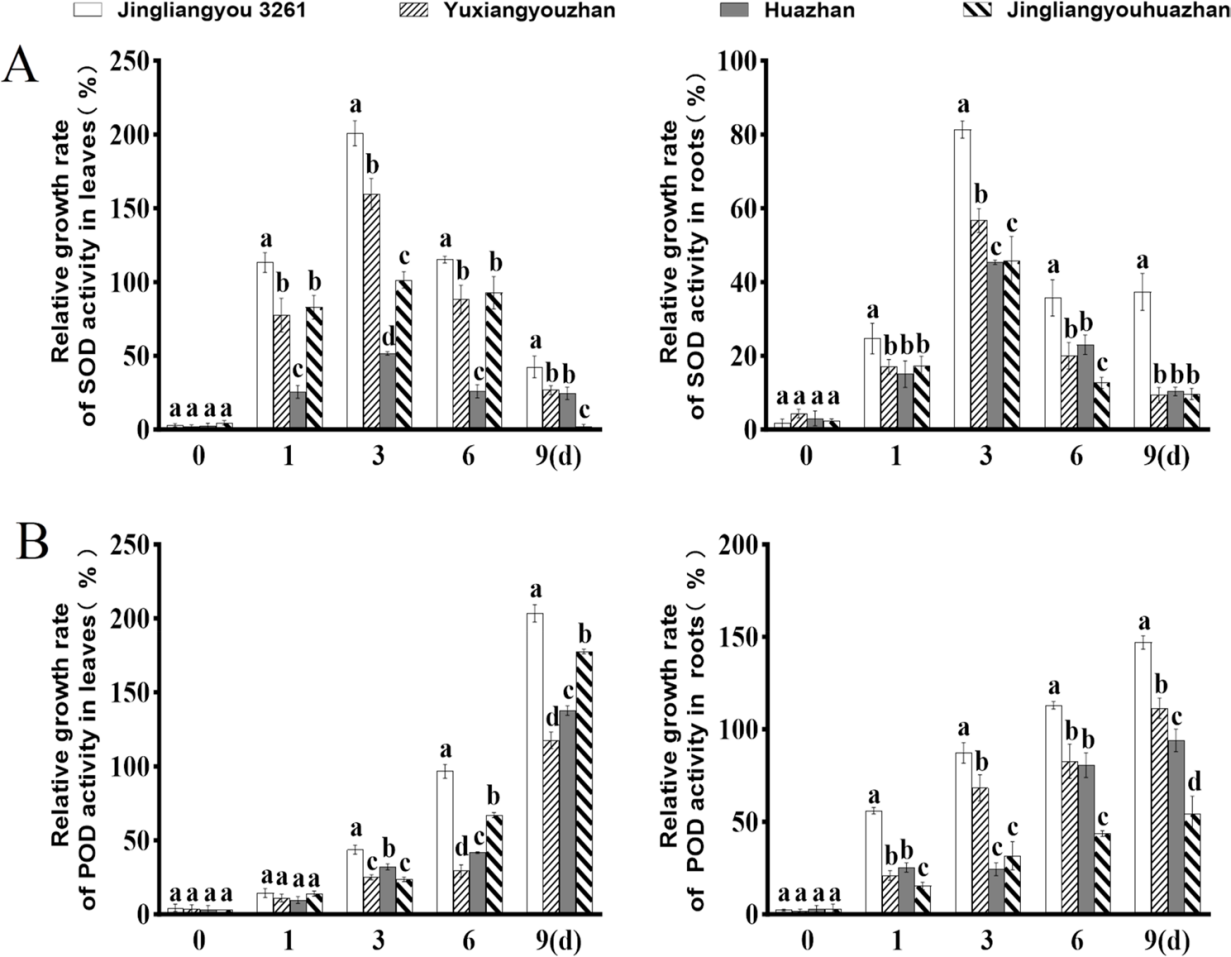
Dynamic Changes in SOD and POD Activities in Leaves and Roots of Contrasting Rice Cultivars Under Salt Stress. **A** SOD activity in leaves and roots of different rice cultivars at 0, 1, 3, 6, and 9 days under salt stress. **B** POD activity in leaves and roots of different rice cultivars at 0, 1, 3, 6, and 9 days under salt stress. Data are presented as mean ± SE of three independent biological replicates (*n* = 3). Different lowercase letters denote significant differences among treatments at each time point (Duncan’s multiple range test, *P* < 0.05)

Salt stress significantly enhanced antioxidant activities activities.SOD activity in both leaves and roots of all cultivars first increased and subsequently declined (Fig. 4A). *Jingliangyou 3261* exhibited the strongest response, with leaf and root activities peaking at 200.85% and 81.43% on day 3, significantly higher than those of the three control cultivars (*P* < 0.05), and maintaining relatively high levels during the later stages. In contrast, the other cultivars showed much lower peak values and a sharp decline thereafter. POD activity increased continuously in all cultivars (Fig. 4B), but *Jingliangyou 3261* again displayed the greatest induction, reaching 203.25% in leaves and 146.79% (relative to the non-stressed control) in roots on day 9, both significantly higher than in the controls (*P* < 0.05). These results demonstrate that, relative to the control cultivars, *Jingliangyou 3261* exhibits enhanced antioxidant enzyme activity and more efficient ROS scavenging capacity under salt stress, thereby reducing oxidative damage and contributing to its superior salt tolerance at the seedling stage.

### Expression profiles of osmotic adjustment genes

*OsP5CS1* and *OsP5CS2* (key proline biosynthesis genes) and *OsLEA3* (encoding a late embryogenesis abundant protein), all important osmotic adjustment genes, were markedly up-regulated in both leaves and roots under salt stress .

**Fig. 5:**
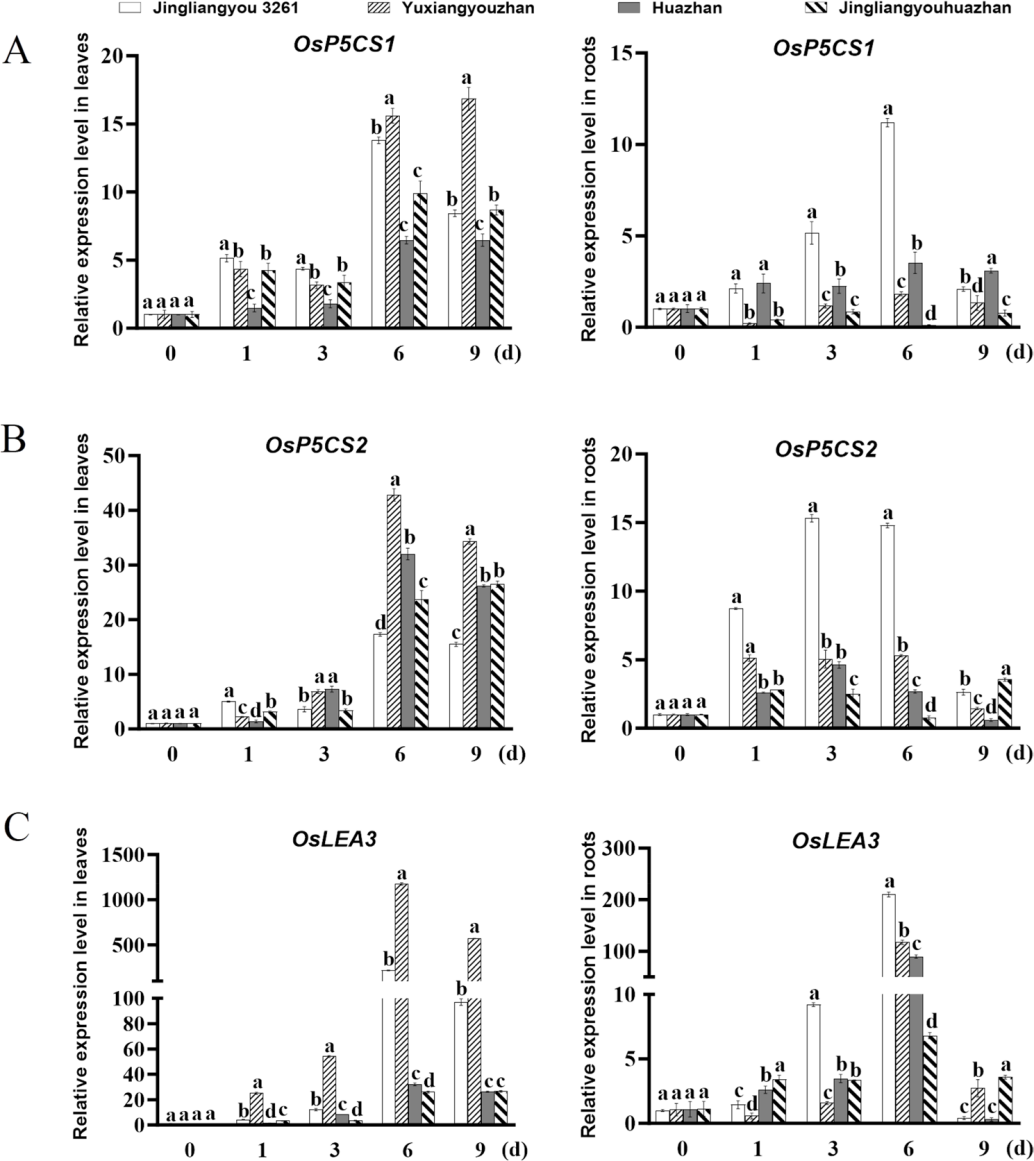
Salt Stress-Induced Expression of *OsP5CS1*,* OsP5CS2* and *OsLEA3* in Different Rice Cultivars. **A** Relative expression level of *OsP5CS1* in leaves and roots of different rice cultivars at 0, 1, 3, 6, and 9 days under salt stress. **B** Relative expression level of *OsP5CS2* in leaves and roots of different rice cultivars at 0, 1, 3, 6, and 9 days under salt stress. **C** Relative expression level of *OsLEA3* in leaves and roots of different rice cultivars at 0, 1, 3, 6, and 9 days under salt stress. Data are presented as mean ± SE of three independent biological replicates (*n* = 3). Different lowercase letters denote significant differences among treatments at each time point (Duncan’s multiple range test, *P* < 0.05)

Under 0.8% NaCl stress, the expression of osmotic adjustment genes showed distinct tissue‑specific and temporal patterns. *OsP5CS1* expression in *Jingliangyou 3261* peaked at day 6, reaching 13.80‑fold in leaves (significantly higher than in *Huazhan* at 6.47‑fold, *P* < 0.01, and *Jingliangyouhuazhan* at 9.73‑fold, *P* < 0.01) and 11.18‑fold in roots (surpassing all controls, *P* < 0.01) (Fig. 5A). *OsP5CS2* also peaked at day 3–6 in leaves (17.30‑fold), lower than the three controls but showing more stable dynamics, while in roots it peaked earlier at day 3 (14.77‑fold), significantly higher than all controls, and remained highest at day 9 (2.63‑fold, *P* < 0.01) (Fig. 5B). *OsLEA3* in leaves peaked at day 6 (218.81‑fold), significantly higher than *Huazhan* (31.42‑fold, *P* < 0.01) and *Jingliangyouhuazhan* (26.00‑fold, *P* < 0.01) throughout days 1–9; in roots, it also peaked at day 6 (209.45‑fold), significantly exceeding all controls (*P* < 0.05) (Fig. 5C). These results indicate that Jingliangyou 3261 activates osmotic adjustment genes more rapidly and sustains higher expression, particularly in roots, contributing to its superior salt tolerance.

### Expression profiles of ion transport genes

Na⁺ and K⁺ transporters were markedly induced in *Jingliangyou 3261*, including *HKT* and *SOS1* families (promoting Na⁺ efflux and xylem unloading) and *NHX* genes (vacuolar Na⁺ sequestration), as well as *HAK* and *KAT* transporters (supporting K⁺ uptake and long-distance transport), together indicating a coordinated strategy to maintain ionic homeostasis under salt stress.

**Fig. 6:**
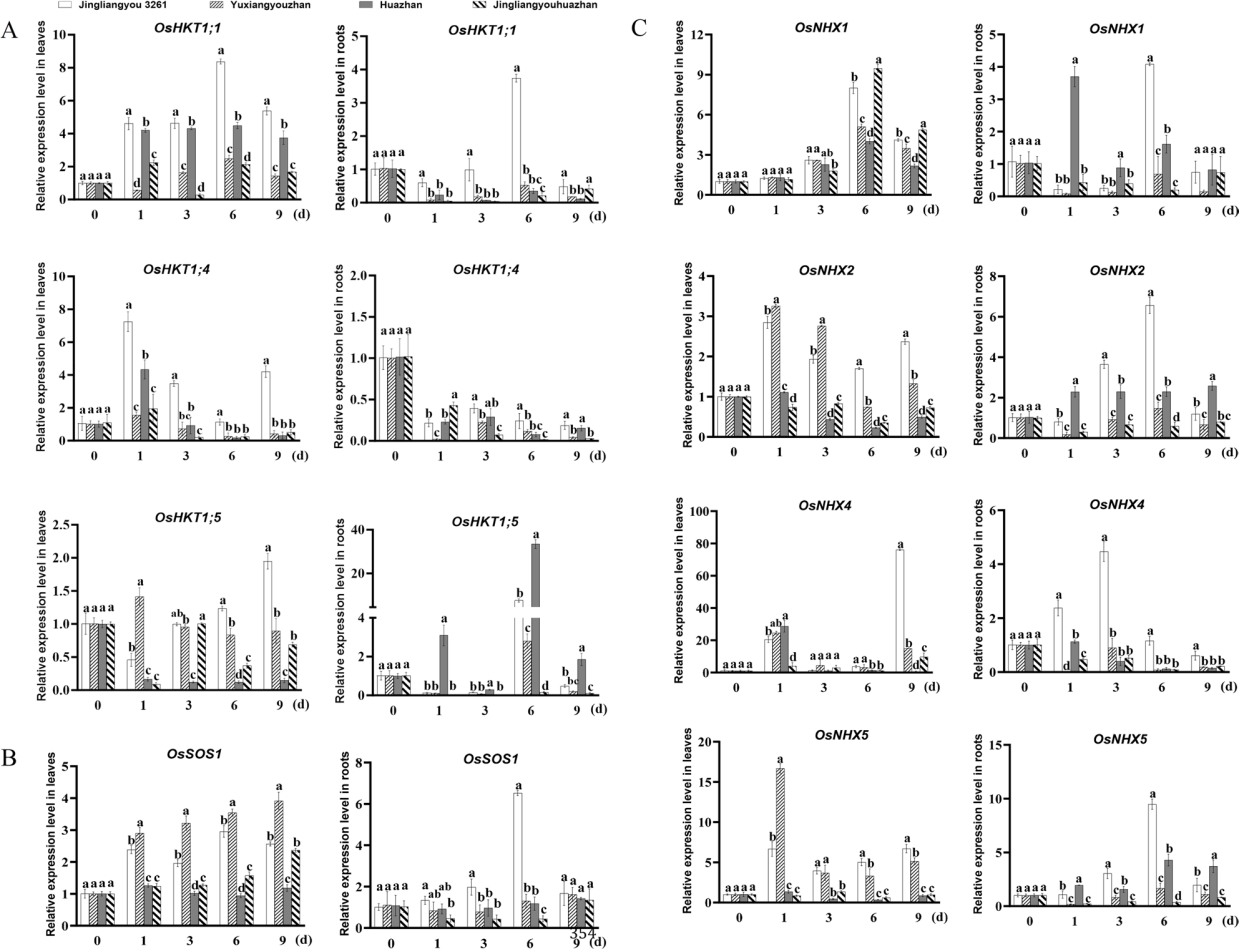
Expression Profiles of Na⁺ Efflux/Sequestration and K⁺ Uptake Transporter Genes in Leaves and Roots of Contrasting Rice Cultivars Under Salt Stress. **A** Relative expression levels of the *HKT* gene family in leaves and roots of different rice cultivars at 0, 1, 3, 6, and 9 days under salt stress. **B** Relative expression level of *SOS* in leaves and roots of different rice cultivars at 0, 1, 3, 6, and 9 days under salt stress. **C** Relative expression levels of the *NHX* gene family in leaves and roots of different rice cultivars at 0, 1, 3, 6, and 9 days under salt stress. Data are presented as mean ± SE of three independent biological replicates (*n* = 3). Different lowercase letters denote significant differences among treatments at each time point (Duncan’s multiple range test, *P* < 0.05)

Under 0.8% NaCl stress, their expression showed distinct tissue-specific and temporal patterns. In leaves, *OsHKT1;1* in *Jingliangyou 3261* peaked at day 6 (8.35-fold), significantly higher than *Yuxiangyouzhan* (2.47-fold, *P* < 0.01) and *Huazhan* (4.49-fold, *P* < 0.01); *OsHKT1;4* peaked at day 1 (6.86-fold), exceeding all controls (*P* < 0.01); *OsHKT1;5* peaked at day 9 (1.93-fold), above controls at days 6–9 (Fig. 6A); *OsSOS1* peaked at day 6 (2.93-fold) but showed no significant difference compared with *Huazhan* and *Jingliangyouhuazhan* from day 1 to day 6 (Fig. 6B); *OsNHX1* peaked at day 6 (7.95-fold), higher than *Yuxiangyouzhan* (5.07-fold, *P* < 0.01) and *Huazhan* (3.98-fold, *P* < 0.01); *OsNHX2*, *OsNHX4*, and *OsNHX5* showed no significant differences compared with the control cultivars. (Fig. 6C); In roots, *Jingliangyou 3261* showed a stronger advantage: *OsHKT1;1* peaked at day 6 (3.70-fold), surpassing all controls at days 1–6 (*P* < 0.01); *OsHKT1;4*, and *OsHKT1;5* showed no significant differences compared with the control cultivars. (Fig. 6A); *OsSOS1* peaked at day 6 (6.48-fold), far exceeding all controls (*P* < 0.01) (Fig. 6B); *OsNHX1* peaked at day 6 (3.81-fold), above all controls (*P* < 0.01); *OsNHX2* peaked at day 6 (6.49-fold), significantly higher than all varieties at days 3–6 (*P* < 0.01); *OsNHX4* showed no significant difference compared with the control cultivars.; *OsNHX5* peaked at day 9 (9.42-fold), above controls (*P* < 0.01) (Fig. 6C).These results demonstrate that *Jingliangyou 3261* coordinately up-regulates multiple ion transport genes, achieving superior Na⁺ exclusion, vacuolar sequestration, and K⁺ retention.

### Expression profiles of antioxidant defense genes

Antioxidant-related genes, including *OsCSD1/2* (superoxide dismutases), *OsCATA/B* (catalases), and *OsAPX1/2* (ascorbate peroxidases), were significantly up-regulated in *Jingliangyou 3261*. Their induction was stronger and more sustained than in the three control cultivars, reflecting enhanced ROS‑scavenging capacity.

**Fig. 7:**
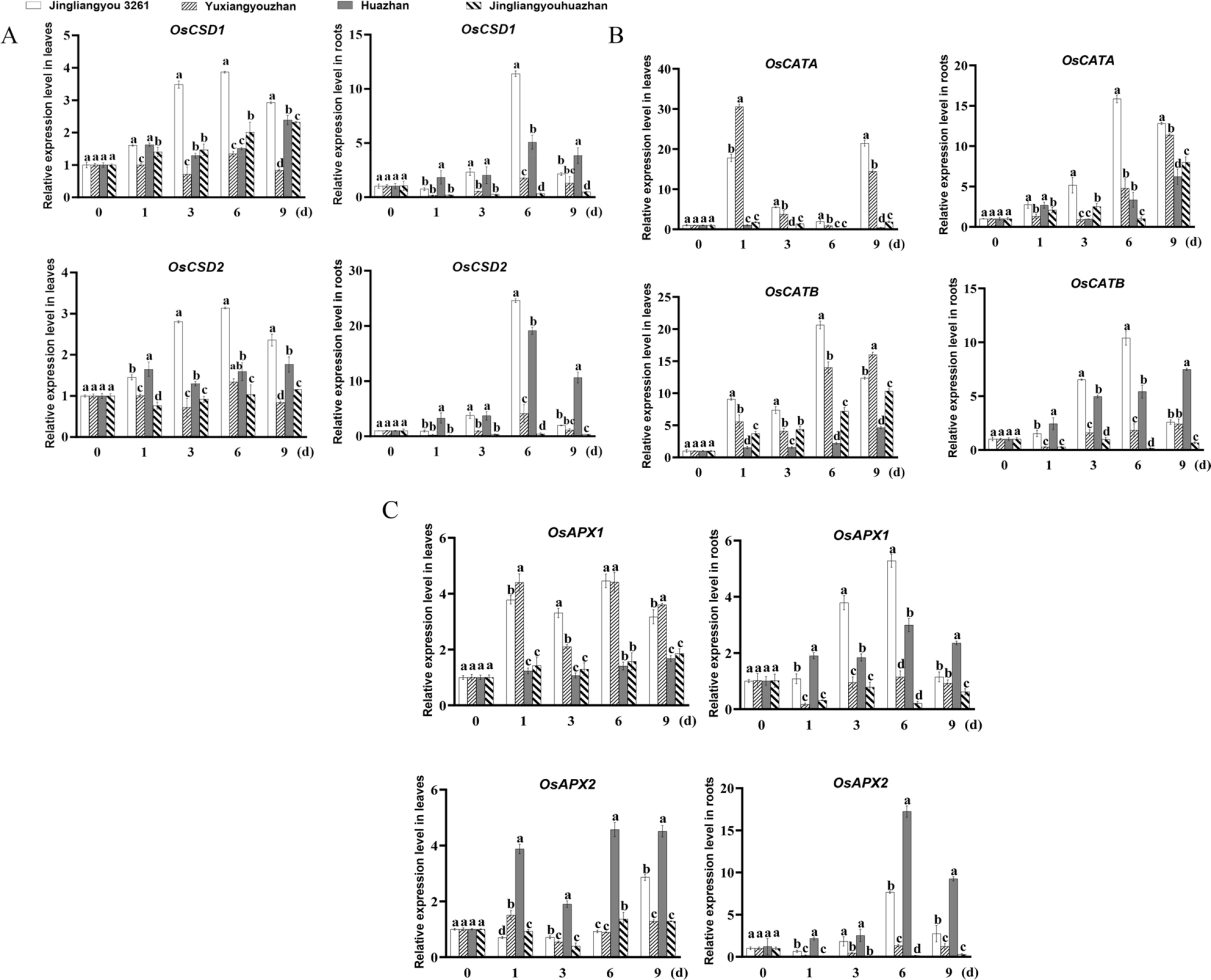
Expression Profiles of ROS-Scavenging Related Genes (*OsCSD1/2*, *OsCATA/B*, *OsAPX1/2*) in Contrasting Rice Cultivars Under Salt Stress. **A** Relative expression levels of *OsCSD1/2* in leaves and roots of different rice cultivars at 0, 1, 3, 6, and 9 days under salt stress. **B** Relative expression levels of *OsCATA/B* in leaves and roots of different rice cultivars at 0, 1, 3, 6, and 9 days under salt stress. **C** Relative expression levels of *OsAPX1/2* in leaves and roots of different rice cultivars at 0, 1, 3, 6, and 9 days under salt stress. Data are presented as mean ± SE of three independent biological replicates (*n* = 3). Different lowercase letters denote significant differences among treatments at each time point (Duncan’s multiple range test, *P* < 0.05)

Under 0.8% NaCl stress, their expression showed distinct tissue‑specific and temporal patterns. For *SOD* genes, leaf expression of *OsCSD1* and *OsCSD2* in *Jingliangyou 3261* peaked at day 6 (3.85‑fold and 3.14‑fold), significantly higher than all controls from day 3 to day 9 (*P* < 0.01). In roots, both genes also peaked at day 6 (11.25‑fold and 24.59‑fold), with significantly higher expression than controls at day 6 (*P* < 0.01) (Fig. 7A). For catalase genes, in leaves, *OsCATA* in *Jingliangyou 3261* was significantly higher than in all control cultivars from day 3 to day 6 (*P* < 0.05); *OsCATB* peaked at day 6 (20.33‑fold), significantly higher than all controls from day 1 to day 6 (*P* < 0.01). In roots, *OsCATA* peaked at day 6 (15.85‑fold) and *OsCATB* peaked at day 6 (10.31‑fold), both significantly higher than the controls (*P* < 0.01) for *OsCATA* at days 3–9 and for *OsCATB* at days 3–6) (Fig. 7B). For *APX* genes: In leaves, *OsAPX1* peaked at day 6 (4.40‑fold), significantly higher than *Huazhan* and *Jingliangyouhuazhan* at days 1–9 (*P* < 0.01); *OsAPX2* peaked at day 9 (2.87‑fold), significantly higher than *Yuxiangyouzhan* and *Jingliangyouhuazhan* at days 3 and 9 (*P* < 0.01). In roots, *OsAPX1* peaked at day 6 (5.28‑fold) and *OsAPX2* at day 6 (7.61‑fold), with *Jingliangyou 3261* showing significantly higher expression than all controls at multiple time points (days 1–9 for *OsAPX2)* (*P* < 0.01) (Fig. 7C). These results demonstrate that *Jingliangyou 3261* activates antioxidant genes more rapidly, strongly, and persistently, particularly in roots, conferring superior oxidative stress tolerance.

Collectively, the phenotypic, physiological, and molecular data demonstrate that *Jingliangyou 3261* seedlings exhibit superior salt tolerance compared with the control cultivars. This advantage is supported by a multi-layered adaptive strategy, including the maintenance of growth and survival under salt stress, preservation of photosynthetic pigments and alleviation of oxidative damage, enhanced accumulation of soluble proteins and improved ion homeostasis achieved via Na⁺ extrusion and K⁺ retention, activation of antioxidant enzyme systems, and robust transcriptional activation of genes related to osmotic adjustment, ion transport, and antioxidant defense. This integrated response highlights the synergistic contribution of physiological and molecular mechanisms to the salt tolerance of *Jingliangyou 3261* at the seedling stage and provides a basis for further discussion of its salt tolerance-related regulatory networks.

## Discussion

### Salt stress reduces growth, but *Jingliangyou 3261* maintains superior morphology and biomass

Salt stress imposes a combination of osmotic stress, ionic toxicity, and oxidative damage on rice seedlings, leading to inhibited growth, chlorosis, and reduced biomass [40, 41]. In this study, under 0.8% NaCl stress, all four tested cultivars (*Jingliangyou 3261*,* Yuxiangyouzhan*, *Huazhan*, *Jingliangyou Huazhan*) showed reduced plant height, root growth, and biomass. However, *Jingliangyou 3261* consistently outperformed the three control cultivars across all measured parameters: it maintained the highest chlorophyll content, the lowest Na⁺/K⁺ ratio, the highest soluble protein content, the lowest MDA levels, and the strongest induction of key salt‑tolerance genes. These results demonstrate that *Jingliangyou 3261* possesses a highly effective, multi‑layered salt tolerance strategy that operates from the molecular to the whole‑plant level.

Furthermore, multi-year official field trials under saline-alkali conditions confirmed the yield advantage of Jingliangyou 3261. In 2021–2022 coastal paddy trials, it achieved an average yield of 354.9 kg per mu, representing a 9.0% increase over control cultivars. Production validation trials further recorded a stable yield of 395.4 kg per mu, a 7.3% increment. The variety also holds an official salt tolerance certification (Grade 5 for full growth period under 3‰ salinity) and is approved for commercial planting in coastal saline-alkali regions with soil salinity below 0.5%. Collectively, these field-level agronomic performances corroborate our laboratory-based mechanistic results, demonstrating that the multi-layered salt-tolerant regulatory network of Jingliangyou 3261 effectively sustains both plant growth and agricultural yield under saline-alkali stress.

### Upstream regulatory networks likely coordinate multi‑pathway activation in *Jingliangyou 3261*

*Jingliangyou 3261* shows stronger and coordinated upregulation of genes from three functional categories: osmotic (*OsP5CS*,* OsLEA3*), ion transport (*OsHKT1;1*,* OsNHX1/4*,* OsSOS1*,* OsHAK*,* OsKAT*), and antioxidant defense (*OsCSD*,* OsCAT*,* OsAPX*). This pattern may reflect enhanced activation of upstream regulatory networks rather than independent promoter action.

Supporting evidence includes: ABA-dependent transcription factors (e.g., *OsDREB2A*) target both *OsP5CS1* and *OsNHX1* [42, 43]; the SOS pathway (*SOS3*-*SOS2*-*SOS1*) induces *OsSOS1* and enhances vacuolar NHX activity, linking Na⁺ exclusion to sequestration [44]; and MAPK cascades (e.g., *OsMAPK5*) regulate ROS scavenging and ion transport [45].

We hypothesize that *Jingliangyou 3261* carries allelic or epigenetic variants in key upstream regulators (e.g., *OsDREB2A* or *OsSOS2*), enabling a faster, stronger transcriptional response to salt stress. Testing this will require comparative time-course transcriptomics between *Jingliangyou 3261* and a sensitive cultivar.

### Enhanced osmotic adjustment, antioxidant defence, and ion homeostasis form a synergistic network

**Fig. 8:**
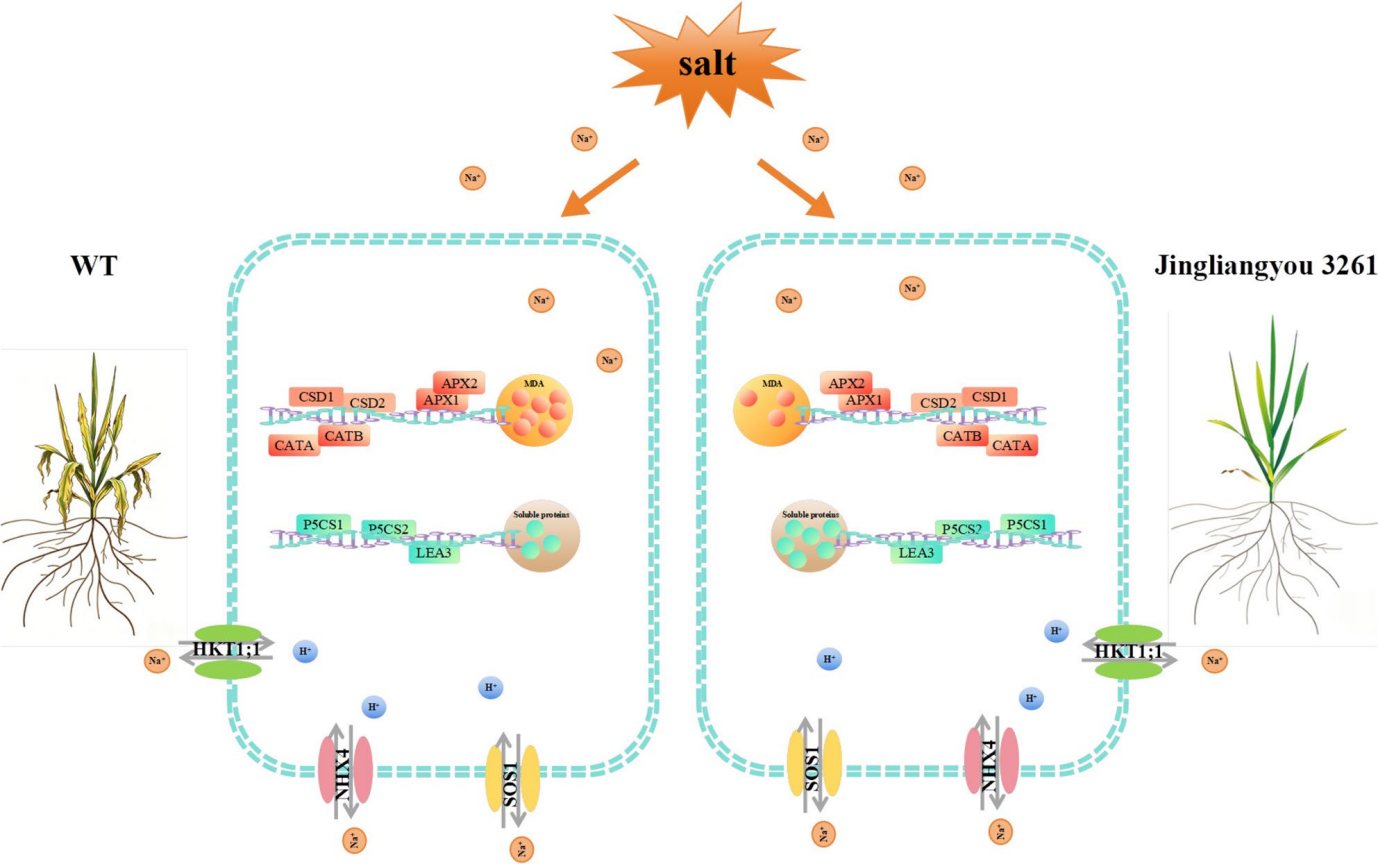
Proposed mechanistic model of salt tolerance at the seedling stage of the hybrid rice *Jingliangyou 3261*

Salt stress lowers soil water potential, causing physiological drought. Plants accumulate osmolytes such as proline and soluble proteins to lower intracellular osmotic potential [46]. In our study, soluble protein content increased in all cultivars, but *Jingliangyou 3261* showed the highest increase, consistent with stronger induction of *OsP5CS1/2* and *OsLEA3*. Proline stabilizes proteins and scavenges ROS [47], while LEA proteins protect against desiccation and ion‑induced aggregation [48]. The higher, sustained expression of these genes correlates with better growth maintenance. Moreover, accumulated soluble proteins support both osmotic adjustment and the preservation of enzyme activities (e.g., SOD, POD) under stress. Importantly, *Jingliangyou 3261* does not rely on a single “super‑gene” but on the coordinated, stronger upregulation of multiple genes across three functional categories: osmotic (*OsP5CS1/2*), ion (especially *OsNHX4*,* OsHKT1;1*, and *OsSOS1*), and antioxidant (*OsCSD1*). This pattern suggests that the upstream regulatory network (e.g., transcription factors like *OsDREB2A* and the SOS signalling pathway) is more efficiently activated in *Jingliangyou 3261* [49]. Based on these findings, we propose a possible mechanistic model diagram (Fig. 8) summarizing the salt‑tolerant physiological characteristics and related gene expression analysis at the seedling stage of the hybrid rice *Jingliangyou 3261*.

### Limitations and future directions

While our study provides a broad analysis, several limitations need to be acknowledged. First, all molecular evidence is at the transcriptional level (qRT‑PCR); protein levels and enzyme activities were not directly measured, so strong *OsNHX4* mRNA induction does not guarantee proportional functional protein. Second, our conclusions are correlative and lack functional validation such as gene overexpression or CRISPR knockout. Third, the experiments were limited to the seedling stage, a single salt concentration (0.8%), and a short duration, whereas salt tolerance mechanisms often differ at reproductive stages. Fourth, the genetic basis of the coordinated response in the hybrid *Jingliangyou 3261* (specific alleles, heterosis, or epigenetics) remains unknown. Fifth, vacuolar Na⁺ sequestration was inferred from lower leaf Na⁺/K⁺ ratios rather than directly measured. Future priorities include: Western blotting or proteomics for key proteins; functional validation via overexpression and CRISPR knockout; field trials at multiple growth stages; and QTL mapping or transcriptomic analysis to dissect the upstream regulatory network, as well as further verification on the causal relationship between gene expression and physiological traits.

## Conclusion

Overall, our findings demonstrate that the superior salt tolerance of *Jingliangyou 3261* seedlings is not attributable to a single trait but rather arises from a synergistic network of morphological adaptation, physiological adjustment, and molecular regulation. This multi-layered response enables *Jingliangyou 3261* to balance osmotic adjustment, ion homeostasis, and oxidative defense more effectively than sensitive cultivars. These results provide valuable insights into the genetic basis of hybrid rice salt tolerance and identify multiple candidate genes that can be exploited for breeding programs targeting saline soils.

## Data Availability

No datasets were generated or analysed during the current study.
